# Comparing video-based versions of Halsted’s ‘see one, do one’ and Peyton’s ‘4-step approach’ for teaching surgical skills: a randomized controlled trial

**DOI:** 10.1186/s12909-020-02105-5

**Published:** 2020-06-17

**Authors:** Lukas B. Seifert, Benedikt Schnurr, Maria-Christina Stefanescu, Robert Sader, Miriam Ruesseler, Jasmina Sterz

**Affiliations:** 1 Department of Oral, Cranio-Maxillofacial, and Facial Plastic Surgery, University Hospital Frankfurt, Goethe University, Theodor-Stern-Kai 7, 60590 Frankfurt, Germany; 2 Department of Pediatric Surgery and Pediatric Urology, University Hospital Frankfurt, Goethe University, Frankfurt, Germany; 3 Department of Trauma, Reconstructive and Hand Surgery, University Hospital Frankfurt, Goethe University, Frankfurt, Germany

**Keywords:** Undergraduate medical skills training, Medical education, Peyton’s 4-step approach, Halsted’s see one do one, Cranio-maxillo-facial surgery

## Abstract

**Background:**

Teaching complex motor skills at a high level remains a challenge in medical education. Established methods often involve large amounts of teaching time and material. The implementation of standardized videos in those methods might help save resources. In this study, video-based versions of Peyton’s ‘4-step Approach’ and Halsted’s ‘See One, Do One’ are compared. We hypothesized that the video-based ‘4-step Approach’ would be more effective in learning procedural skills than the ‘See One, Do One Approach’.

**Methods:**

One-hundred-two naïve students were trained to perform a structured facial examination and a Bellocq’s tamponade with either Halsted’s (*n* = 57) or Peyton’s (*n* = 45) method within a curricular course. Steps 1 (Halsted) and 1–3 (Peyton) were replaced by standardized teaching videos. The performance was measured directly (T1) and 8 weeks (T2) after the intervention by blinded examiners using structured checklists. An item-analysis was also carried out.

**Results:**

At T1, performance scores significantly differed in favor of the video-based ‘4-step Approach’ (*p* < 0.01) for both skills. No differences were found at T2 (*p* < 0.362). The item-analysis revealed that Peyton’s method was significantly more effective in the complex subparts of both skills.

**Conclusions:**

The modified video-based version of Peyton’s ‘4-step Approach’ is the preferred method for teaching especially complex motor skills in a large curricular scale. Furthermore, an effective way to utilize Peyton’s method in a group setting could be demonstrated. Further studies have to investigate the long-term learning retention of this method in a formative setting.

## Background

Over the last two decades, many national and international studies have shown that the training of clinical skills in medical and surgical education [1, 2] is insufficient, despite the proven importance of these skills for every medical doctor. Since the clinical routine of young doctors often lacks the time to deal with these deficits, a sound training of clinical skills must have already taken place at the undergraduate level.

Traditionally, clinical skills training in medicine can be summarized under the adage ‘See One, Do One, Teach One’, meaning that trainees, after observing a particular procedure once, are expected to be capable of performing that procedure followed by being able to teach another trainee how to conduct that procedure [3]. Due to the often lack of time resources, the important last step “Teach one” is often omitted or takes part without supervision in everyday clinical practice. Because of the increased awareness for patient safety today, many argue that this teaching method is passé because students are unable to safely perform a medical procedure after seeing it only once [4–6].

Among other instructional approaches, like mental training [7] or the use of teaching associates [8], the ‘4-step Approach’ outlined by Rodney Peyton (1998) has become increasingly popular in teaching clinical competence and in particular procedural skills [9]. The ‘4-step Approach’ consists of the following steps:

1. Demonstration – The teacher performs the skill in real-time without any explanation
2. Deconstruction – The teacher performs the skill slowly, explaining every single step
3. Comprehension – The student explains whereupon the teacher performs every single step of the procedure
4. Execution – The student explains and simultaneously performs every step of the procedure

Originally developed to teach procedural skills in the operating theatre, today, the ‘4-step Approach’ is used as an instructional approach in basic resuscitation and trauma management [10–12], and has also been shown to be effective in the instruction of basic procedural skills, such as surgical suturing [13, 14] or intravenous cannulation [15, 16]. Krautter et al. identified Step 3 as the most crucial part of Peyton’s ‘4-step Approach’, contributing significantly more to the learning success than the previous steps due to its inherent combination of motor imaginary [17] and skill performance [15]. However, the method is relatively time- and material-consuming and personnel-intensive, and several other studies have shown that simple procedural skills can also be taught with a considerably reduced didactic effort [18, 19], which raises the question of how and for which procedural skill, in particular, Peyton’s ‘4-step Approach’ should be used. For example, Orde et al. could show that Peyton’s ‘4-step Approach’ is not superior compared to the ‘See One, Do One’ approach in learning how to place a laryngeal mask. Another similar study by Greif et al. could find no superiority of the Peyton’s ‘4-step Approach’ in the learning of an emergency cricothyroidotomy compared to the ‘See One, Do One’ approach.

A possible way to reduce material and personal recourses is to modify the ‘4-step Approach’ by implementing standardized educational videos [20]. Previous studies have demonstrated that replacing steps 1 and 2 through standardized educational videos does not have any negative effects on the learning outcomes compared to other teaching methods [11]. However, it remains unclear how many steps of the ‘4-step Approach’ can be replaced yet remaining effective in practical skills training, and there is no standardized protocol to use this method – that was designed for individual skills training – during small group teaching. For this reason, in this study, we examine a newly modified video-based ‘4-step Approach’ that replaces steps 1 to 3 with standardized educational videos and compared this to a video-enhanced version of Halsted’s traditional ‘See One, Do One’ Approach in short- and long-term practical skills acquisition.

The hypothesis of our study was that the modified ‘4-step Approach’ was equally or more effective in the acquisition of basic surgical skills from the Cranio and Maxillofacial Surgery (CMF) spectrum as a video-enhanced version of Halsted’s ‘See One, Do One’ Approach in short- and long-term.

## Methods

### Study design

This study is a quasi-randomized controlled educational research study comparing the effects of two different instructional approaches on the acquired practical skills during undergraduate surgical training.

### Study participants

Study participants were undergraduate medical students at Goethe-University Hospital in Frankfurt in the fourth year of a 6-year program completing their obligatory surgical training. All participants were naïve regarding CMF-specific practical skills and knowledge.

Participation was voluntary and took place after written informed consent. Students were blinded in relation to their knowledge of the didactic principles used during their training as well as affiliation to any study group. Basic data regarding student age, sex, and duration of the study were collected using a questionnaire.

The study was conducted according to the ethical principles of the Helsinki Declaration (Ethical Principles for Medical Research Involving Human Subjects), and the local ethics committee noted that no further approval was necessary.

### Study protocol

The study was carried out within the ‘training week of practical clinical skills in surgery’ [21]. This week aims to teach students basic surgical competencies and prepare them for their upcoming surgical rotations. This takes place at the skills lab and consists of 12 teaching units for basic general and surgical skills from all surgical disciplines. The teaching content is based on the learning objectives for practical skills defined in the national competency-based catalog of learning objectives in surgery from the German Society of Surgery [22]. The training has a capacity of 64 students per week, with a maximum of eight students per group and tutor.

Following the first week of practical skills training, students pass through two weeks of surgical rotation and participate on the ward, the ambulance or in the operating theatre to practice the acquired surgical knowledge and skills under supervision. In the two weeks of surgical rotation no additional CMF-specific training took place since only around 1 to 2% of students passed their surgical rotation at the Department of Cranio-Maxillofacial and Facial Plastic Surgery and therefore did not further practice the investigated skills of this study.

**Fig. 1:**
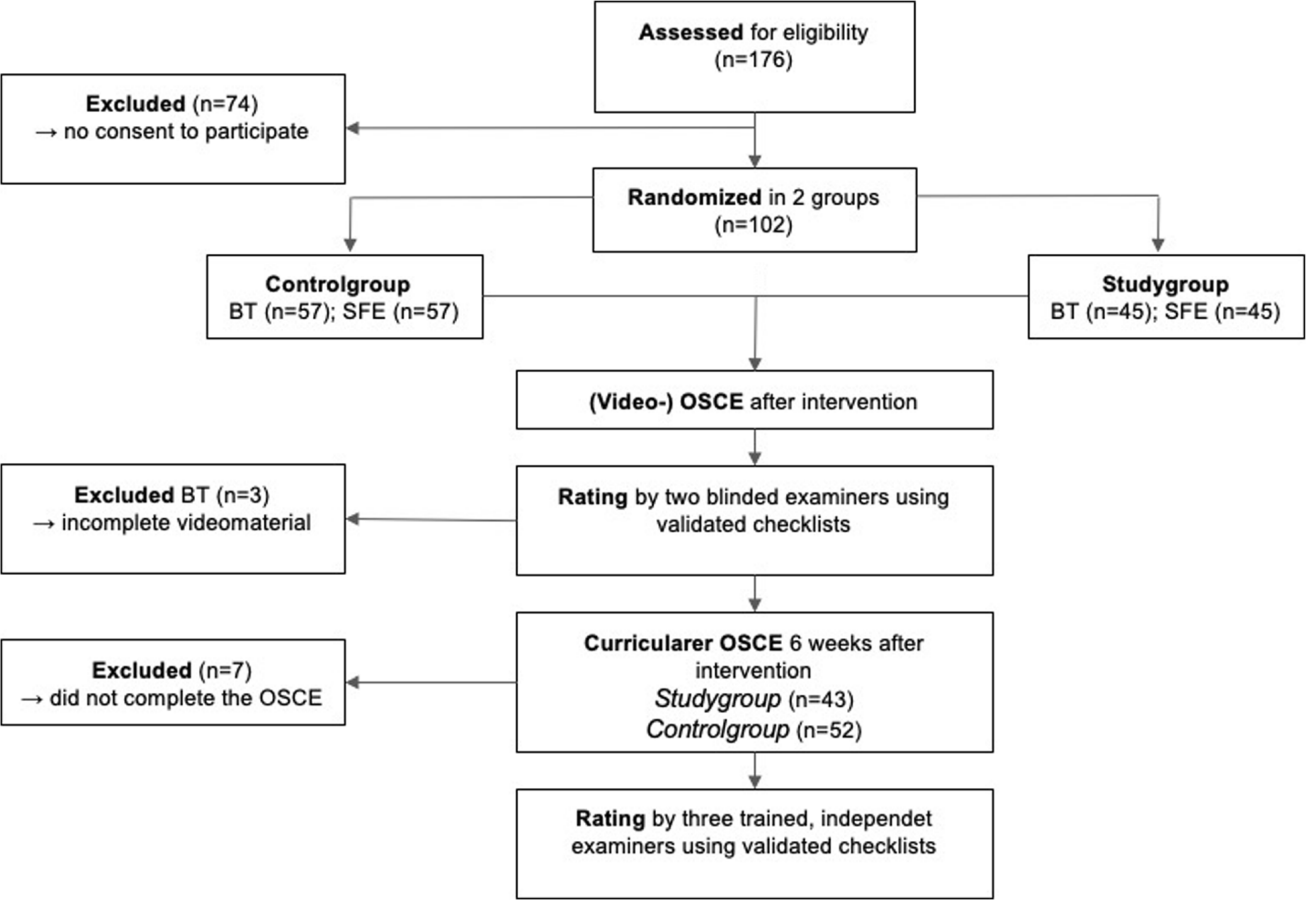
Study design and execution

Two fundamental clinical competences from the CMF spectrum, namely the performance of a structured facial examination (SFE, video at https://youtu.be/S-b3kIzmLQw, accessed 17.04.2020) and the packing of a modified Bellocq tamponade (BT, video at https://youtu.be/gtMq4044RlM, accessed 17.04.2020) were examined (Fig. 1). These competencies were chosen since previous studies found medical students to have significant shortcomings in CMF-specific competencies [23–25], although consultations in the field are of considerably socioeconomic and numerical importance in the acute and normal care [26, 27].

The existing instructional manual for the CMF unit was reworked and adapted for the study. Each manual consists of a detailed schedule and workflow, as well as a step-by-step checklist to ensure a standardized sequence of the training. For further quality assurance and standardization, an instructional video was designed for both skills [28]. All instructors involved received an online-training, where both skills were demonstrated and trained in each of the instructional approaches as well as the correct performance of each instructional approach.

### Intervention

The assignment of students to one of the eight learning groups per training week with a maximum of six students per group who pass through the teaching units together occurred prior to the training week, independent of the authors and independent of study participation by the deanery. The allocation of the learning group in the study to the two instructional approaches was performed alternately.

#### ‘Video See One, Do One Approach’

The ‘See One, Do One Approach’ is one of the main components of clinical-bedside teaching. During the approach students first watch an expert demonstrate and explaining a certain skill which is followed by the first independent performance of the skill, often directly with a patient [3]. To ensure a high standardization for the demonstration, each skill was videotaped based on the existing manual and checklist. First, the trainer demonstrated the video and explained the skill in detail, step-by-step as predetermined in the manual and trained in the tutor training. Subsequently, students could practice the skill under the supervision on peers (SFE) or on training models (BT) and, if needed, receive correction from the tutor. Each student should practice each skill at least once. The training lasted for 60-min.

#### ‘Video 4-step Approach’

For this study, the ‘4-step Approach’ – as described by Walker and Peyton [9] – was modified. For Step 1, the video was demonstrated without any comments by the trainer. For Step 2, the video was demonstrated, and the trainer explained the skill in detail as predetermined in the manual and trained in the online-tutorial. For Step 3, the video was paused after each step, and students were chosen one-by-one by the trainer to explain the next instructional step of the video, which was then video-played. Step 4 was performed, as described by R. Peyton. Here, students explained every upcoming step of the procedure and after approval by the trainer carried it out under supervision.

Subsequently, students could practice the skills under the supervision and, if needed, receive correction from the tutor. Each student was advised to practice each skill at least once. The training lasted for 60-min.

### Outcome measures

To assess the acquired competence in both skills of the study, the OSCE-format (one station for each skill) was chosen during the training week directly after the intervention (T1) and 5–13 (SD = 3.16) weeks later (T2), as part of the curricular and summative surgical OSCE. The surgical OSCE consists of 10 stations in total, two of them regarding the structured facial examination and the packing of a Belloq’s tamponade. Each checklist contained a trinary scoring scale (0 points for not done, 1 point for done but incorrect, and 2 points for done and correct), which was based on the checklist used in the tutor manual. To complete each OSCE station a timeframe of 5-min was given. After performance completion, students received short feedback and suggestions for improvement at T1. During their training week, students were video-recorded (Camera System: Panasonic HC-X929) for later performance measurement. Due to data protection reasons the videos were blinded regarding the student names and were deleted after analysis. The checklists implemented were primarily piloted in previous undergraduate trainings and afterwards validated by two independent, blinded examiners. Therefore, inter-rater reliability was measured using Pearson’s correlation coefficient (r). In addition, the content validity was ensured through the creation as part of an expert workshop with didactic and surgical experts as well as through the repeated application and adaption in the context of previous studies [7, 27, 28] and OSCE exams. During their surgical OSCE, due to examination regulations, the performance was not video-recorded and measured by only one examiner for each skill using the same checklists. Examiners were two experienced surgeons working in CMF surgery. All examiners were blinded towards the students’ instructional approach and affiliation of the learning group. They received training before the OSCE to gain experience in the use of the checklist.

### Data analysis

Microsoft Excel 2016 (Microsoft Office 2007,© Microsoft Corporation, Redmond, USA) for Mac and SPSS Statistics version 19 (IBM, Armonk, USA) were used for the statistical analysis and graphical display of data.

To test for normal distribution the Kolmogorov-Smirnov test for one sample was carried out. Since the data was not normally distributed the Mann-Whitney-U-Test for non-parametric data was used to test for significant differences in learning success between the ‘Video 4-step Approach’ and ‘See One, Do One Approach’. To test for performance differences within the respective groups at different times, the Friedman test for repeated measures was used. Furthermore, effect sizes were calculated using eta squared (η^2^), which is defined as the square of the correlation ratio (η), resulting in a unitless value that helps to interpret the effect size of observed results and hence the statistical power of a study. For most types of effect sizes, a larger absolute value indicates a stronger effect. As a rule of thumb values ≤0.01 indicate a relatively small effect, values ≤0.06 indicate a medium effect size and values ≥0.14 indicate a strong effect. Furthermore, it can be used as an additional control test since prior studies have shown that significant test results alone are not sufficient to interpret data and draw conclusions [29].

Pearson’s correlation coefficient (r) was used to calculate the inter-rater reliability between both raters at T1.

Additionally, each item of the checklists that were used to measure student performance was categorized into four (extraoral examination, neurological examination, midface examination, intraoral examination) or three (material preparation, insertion of the tamponade, fixation of the tamponade) subgroups. Averages from those subgroups were checked for significant differences using the Mann-Whitney-U-Test for non-parametric data.

### Sample size estimation

Based on prior examination results from the years before the intervention, we estimated an average student performance of 70% with a standard deviation of 10% in the OSCE. A sample size of 88 (44 per study arm) was calculated based on the following parameters: mean ‘4-step Approach’ = 33, mean ‘See One, Do One’ = 30, SD = 10, alpha = 0.05, beta = 0.2, Power = 80%.

## Results

### Study participants

One-hundred-two (f = 62; m = 40, age = 23,5 ± 1,7) out of 176 students agreed to participate in the study. Fifty-seven students (m = 18, w = 39, age = 23,6 ± 1,8) were assigned to the ‘See One, Do One’ group while 45 students (m = 22, w = 23, age = 23,4 ± 1,7) were trained using the video-based ‘4-step Approach’. Due to incomplete video footage in the performance of a Bellocq’s tamponade, two students that were trained with the video-based ‘4-step Approach’ and one student that belonged to the ‘See One, Do One’ group had to be excluded from the study. Ninety-five students (f = 56; m = 39) participated in the curricular OSCE 5–13 weeks after the intervention. Seven students did not participate in this OSCE due to illness or other reasons.

Both teaching interventions were carried out in a curricular setting in the given timeframe without any complications.

### Structured facial examination

**Fig. 2:**
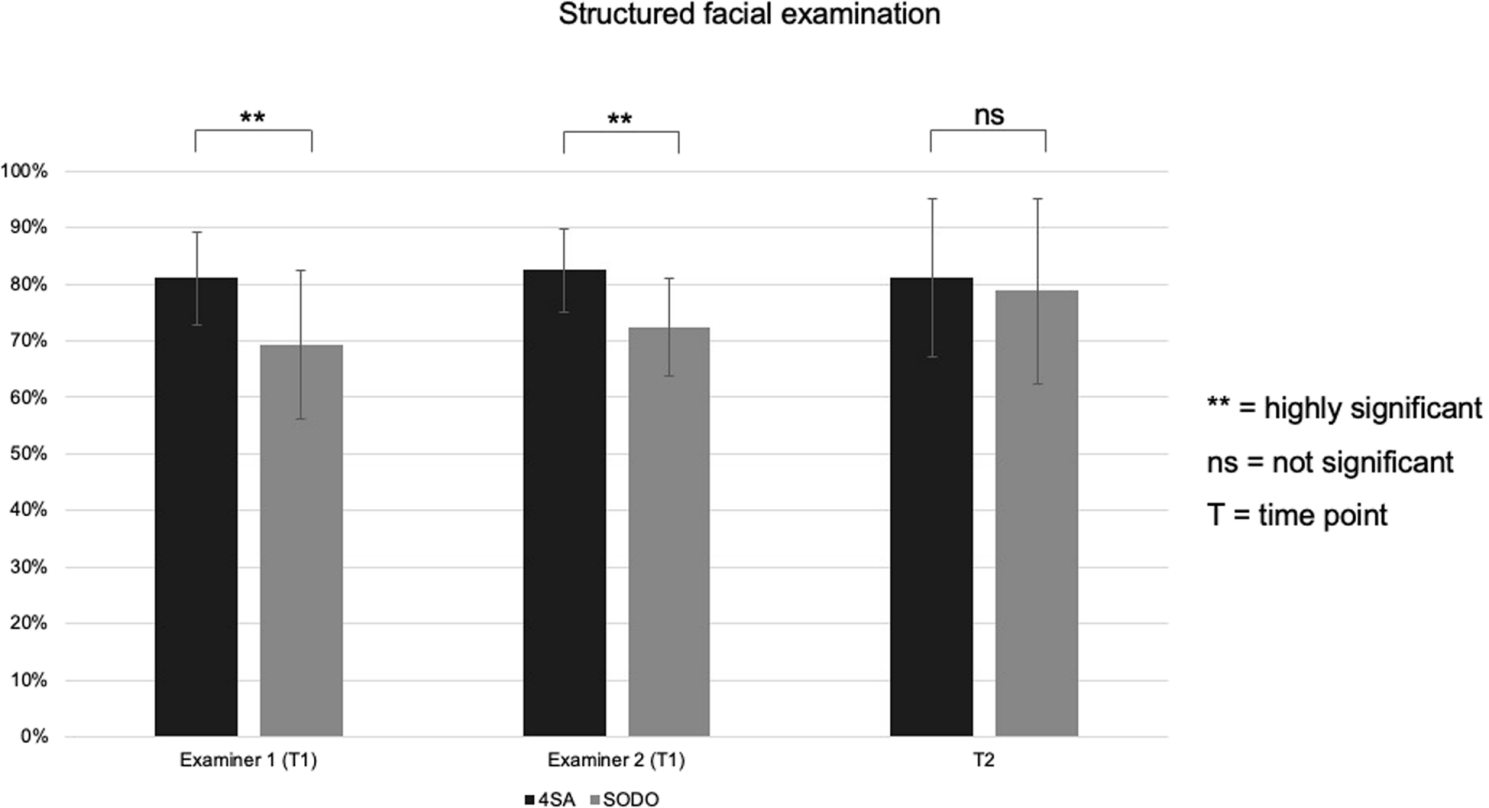
Results on performing a SFE at post-training (T1 from examiner 1 and examiner 2) and retention (T2). Values are presented as mean and the standard deviation (±). * *p* < 0.05, ** *p* < 0.001, n.s. = not significant

At T1, students that were trained with the video-based ‘4-step Approach’ showed highly significantly better results in the performance of a structured facial examination (*p* < 0.001) than students that were trained with the ‘See One, Do One’ approach (Table 1). At T2, no significant difference could be found between both groups (*p* < 0.616, η^2^ = 0.0055). Most (81.1%) of those in the video-based ‘4-step Approach’ group showed very good retention of the acquired knowledge, while the ‘See One, Do One’ group was able to significantly increase its performance by at least 6.4% compared to the first measure (Fig. 2).

**Table 1:** Examination results of the video-based ‘4-step Approach’ group (4SA) and the ‘See One, Do One’ (SODO) group at T1 and T2 for performing a structured facial examination. Shown in the table are the means in percent, the standard deviation (±) in percent and the confidence interval (CI)

	Examiner 1 (post-training)	Examiner 2 (post-training)	retention
**4SA**	81.1% (± 8.2%; CI = 78.6–83.4)	82.5% (± 7.3%; CI = 79.8–84.1)	81.2% (± 14.0%; CI = 76.9–85.0)
**SODO**	69.3% (± 13.1%; CI = 65.6–72.4)	72.4% (± 8.7%; CI = 69.7–74.2)	78.8% (± 16.4%; CI = 73.7–82.2)
***p*-Value**	***p*** **< .001**	***p*** **< .001**	*p* < .616
**η**^**2**^	0.2235	0.2825	0.0055

The item-based analysis revealed significant differences in favor of the video-based ‘4-step Approach’ in all three subgroups (‘neurological examination’, ‘examination of the midface’ and ‘intraoral examination’) at T1 (Table 2). At T2, no significant differences could be found between the groups. The inter-rater correlation (0.585) between both examiners at T1 was satisfactory.

**Table 2:** Item-based analysis of the examination results between the video-based ‘4-step Approach’ group (4SA) and the ‘See One, Do One’ (SODO) group at T1 and T2. Significant differences in favour of the video-based ‘4-step Approach’ group could be found in the subgroups ‘neurological examination’, ‘midface examination’ and ‘intraoral examination’. Shown in the table are the means in percent, the standard deviation (±) in percent and the confidence interval (CI)

		Extraoral Examination	Neurological Examination	Midface Examination	Intraoral Examination
**4SA**	Examiner 1 (post-training)	32.2% (± 41.0%; CI = 20.0–43.9)	89.1% (± 12.3%; CI = 85.4–92.5)	87.0% (± 11.6%; CI = 83.6–90.3)	63.9% (± 10.6%; CI = 59.9–66.1)
	Examiner 2 (post-training)	35.6% (± 45.6%; CI = 21.6–48.3)	94.2% (± 16.7%; CI = 89.1–98.8)	86.3% (± 11.2%; CI = 82.7–89.2)	67.2% (± 11.3%; CI = 63.7–70.3)
	retention	75.0% (± 25.0%; CI = 67.7–82.3)	91.0% (± 14.9%; CI = 86.6–95.3)	77.9% (± 19.5%; CI = 71.3–82.7)	81.3% (± 19.1%; CI = 75.4–86.5)
**SODO**	Examiner 1 (post-training)	24.6% (± 36.4%; CI = 14.5–33.4)	81.6% (± 15.2%; CI = 77.0–84.9)	71.6% (± 15.8%; CI = 66.9–75.1)	57.9% (± 17.9%; CI = 52.3–61.6)
	Examiner 2 (post-training)	24.6% (± 38.8%; CI = 13.9–34.0)	92.0% (± 10.3%; CI = 89.3–94.6)	73.8% (± 12.5%; CI = 69.7–76.2)	53.8% (± 31.2%; CI = 44.9–61.1)
	retention	68.3% (± 24.1%; CI = 64.3–71.6)	92.5% (± 10.5%; CI = 89.2–94.7)	74.6% (± 21.5%; CI = 68.4–79.5)	78.4% (± 26.8%; CI = 71.0–84.9)
***p*-Values**	Examiner 1 (post-training)	*p* < .435	***p*** **< .008**	***p*** **< .001**	*p* < .138
	Examiner 2 (post-training)	*p* < .332	*p* < .107	***p*** **< .001**	***p*** **< .001**
	retention	*p* < .267	*p* < .968	*p* < .477	*p* < .764

### Bellocq’s tamponade

**Fig. 3:**
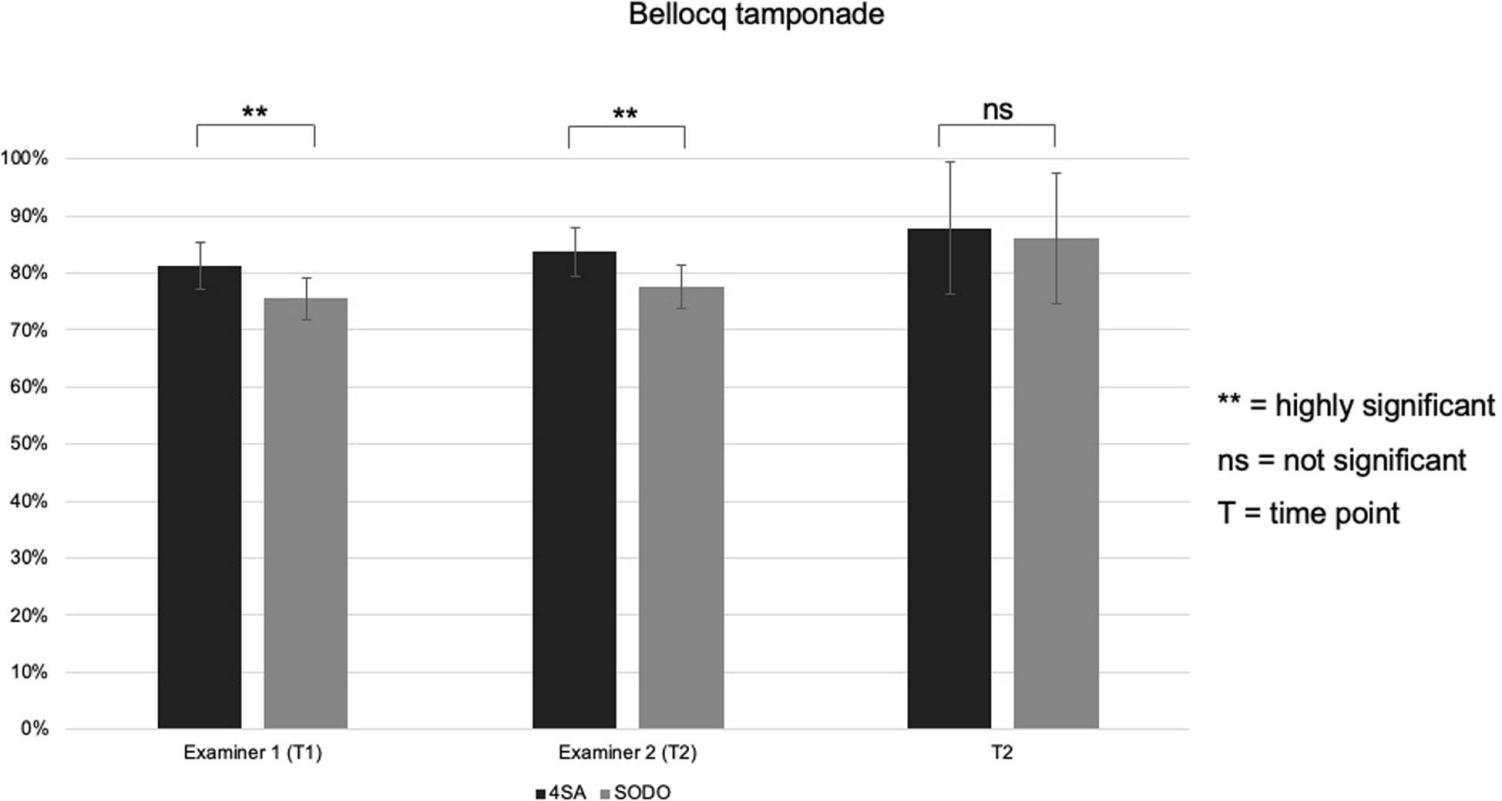
Results on performing a BT at post-training (T1 from examiner 1 and examiner 2) and retention (T2). Values are presented as mean and the standard deviation (±). * *p* < 0.05, ** *p* < 0.001, n.s. = not significant

At T1, students that were trained with the video-based ‘4-step Approach’ showed highly significantly better results in the performance of a modified Bellocq’s tamponade (*p* < 0.001) than students that were trained with the ‘See One, Do One’ approach. At T2, no significant difference could be found (*p* < 0.362, η^2^ = 0.0067). Both groups were able to significantly increase their performance from T1 to T2 (Table 3; Fig. 3).

**Table 3:** Examination results of the video-based ‘4-step Approach’ group (4SA) and the ‘See One, Do One’ (SODO) group at T1 and T2 for the insertion of a modified Bellocq’s tamponade. Shown in the table are the means in percent, the standard deviation (±) in percent and the confidence interval (CI)

	Rater 1 (post-training)	Rater 2 (post-training)	retention
**4SA**	81.3% (± 4.4%; CI = 79.7–82.2)	83.7% (± 5.5%; CI = 81.3–84.6)	87.9% (± 11.5%; CI = 83.6–90.3)
**SODO**	75.5% (± 8.0%; CI = 72.9–77.0)	77.6% (± 8.4%; CI = 74.8–79.1)	86.2% (± 11.5%; CI = 83.0–88.9)
***P*-Value**	**p < .001**	***p*** **< .001**	*p* < .362
**η**^**2**^	0.1703	0.1545	0.0067

The item-based analysis revealed significant differences in favor of the video-based ‘4-step Approach’ in the subgroups ‘material preparation’ and ‘insertion of the tamponade’, but no significant differences in the subgroup ‘fixation of the tamponade’ at T1. At T2, no significant differences could be found for any subgroup (Table 4). With 0.104, the inter-rater correlation between both examiners at T1 was rather weak.

**Table 4:** Item-based analysis of the examination results between the video-based ‘4-step Approach’ group (4SA) and the ‘See One, Do One’ (SODO) group at T1 and T2. Significant differences in favor of the video-based ‘4-step Approach’ group could be found in the subgroups ‘material preparation’ and ‘catheter insertion’. No differences were found at T2. Shown in the table are the means in percent, the standard deviation (±) in percent and the confidence interval (CI)

		Material preparation	Catheter Insertion	Tamponade Fixation
**4SA**	Examiner 1 (post-training)	83.3% (± 3.6%; CI = 81,9–84.0)	96.6% (± 6.0%; CI = 94.2–97.7)	58.3% (± 14.0%; CI = 53.9–62.0)
	Examiner 2 (post-training)	94.3% (± 10.0%; CI = 91.0–96.9)	98.3% (± 4.3%; CI = 96.7–99.2)	53.4% (± 18.3%; CI = 47.6–58.3)
	retention	88.1% (± 20.7%; CI = 81.9–94.0)	98.2% (± 4.4%; CI = 96.7–99.2)	73.8% (± 25.2%; CI = 65.6–80.3)
**SODO**	Examiner 1 (post-training)	71.5% (± 19.0%; CI = 66.5–76.4)	91.8% (± 9.6%; CI = 88.5–93.4)	57.6% (± 11.4%; CI = 54.0–59.9)
	Examiner 2 (post-training)	75.2% (± 21.5%; CI = 69.4–80.5)	94.5% (± 7.8%; CI = 91.2–96.7)	57.3% (± 13.4%; CI = 53.5–60.4)
	retention	93.0% (± 12.5%; CI = 89.7–96.2)	93.5% (± 10.7%; CI = 90.2–95.7)	69.7% (± 25.3%; CI = 62.4–75.5)
***p*-value**	Examiner 1 (post-training)	***p*** **< .003**	***p*** **< .011**	*p* < .756
	Examiner 2 (post-training)	***p*** **< .001**	***p*** **< .033**	*p* < .631
	retention	*p* < .596	*p* < .057	*p* < .322

## Discussion

The aim of this study was to investigate the teaching efficacy of two teaching methods in the short- and long-term acquisition of two surgical skills, namely the structured facial examination and the insertion of a Bellocq’s tamponade: a video-based ‘4-step Approach’ and the ‘See One, Do One Approach’. Overall, our results revealed significant performance differences between both groups in the short-term examination (T1) in favor of the ‘4-step Approach’. Furthermore, the re-examination 5–13 weeks later (T2) revealed a very good long-term learning retention of the acquired practical skills for the group that has been taught using the ‘4-step Approach’. Students of the ‘See One, Do One’ group, however, were able to significantly improve their level of competence to the level of the ‘4-step Approach’ group in the long-term comparison. The implementation of the video-based ‘4-step Approach’ in a curricular setting was completely feasible within the given timeframe of the ‘training week of practical clinical skills in surgery’ [21].

We believe a reason for that is Step 3 of the ‘4-step Approach’ namely, the verbalization and subsequent instruction of a complex motor skill, led to a more profound cognitive processing and hence, to a better skills performance of the ‘4-step Approach’ group compared to the ‘See One, Do One’ group. This presumption is also supported by a previous study by Krautter et al., who identified Step 3 as the most crucial part of Peyton’s original ‘4-step Approach’ [15]. These authors assumed that the reason for this is that it combines motor imagery [30] and skills performance and is hence superior to skills observation-only, like in the ‘See One, Do One Approach’.

Interestingly, the ‘See One, Do One’ group was able to significantly improve its performance in the long-term examination at T2 while the ‘4-step Approach’ group managed to maintain a high-performance level even though other studies found a clear benefit for Peyton’s ‘4-step Approach’ compared to other teaching formats in the long-term comparison [16]. Hermann-Werner et al. compared the use of a ‘best practice-model’ containing structured individual feedback and Peyton’s ‘4-step Approach’ to the traditional ‘See One, Do One Approach’ for teaching the insertion of a nasogastric tube and i.v. cannulation. These authors found Peyton’s ‘4-step Approach’ to be superior both in the short- and long-term comparison. However, their study was conducted in a non-curricular ‘in vitro’ setting within a skills-laboratory [16]. In the present study, the performance assessment at T2 as part of a curricular and formative surgical OSCE approach might have been influenced by the desire of all participating students to perform well. This could have led to the significant performance improvement of the ‘See One, Do One’ group when measured in the long-term. This phenomenon is also known as “assessment drives learning” and has been described in detail by Raupach et al., who found that summative examinations to be ‘more powerful drivers of student learning than the instructional format’ itself [31].

When looking closer at the results of this study, it is noticeable that the performance differences and effect sizes between the ‘4-step Approach’- and the ‘See One, Do One’ group were greater for the performance of an SFE compared to the insertion of a Bellocq’s tamponade. This difference can be explained with the higher level of complexity of the SFE compared to the Bellocq’s tamponade (Supplements 1 and 2) since previous studies found a clear benefit of Peyton’s ‘4-step Approach’ for very challenging motor skills, such as laparoscopic suturing and knot tying [14] or the replacement of a complex wound dressing or performing a simple interrupted suture [20]. For relatively easy to learn motor skills like performing external chest compressions or the placement of a laryngeal tube, no significant advantages could be found for Peyton’s ‘4-step Approach’ in previous studies (Orde 2010; Münster 2016). The results of the item-based analysis support the assumption that the ‘4-step Approach’ is particularly useful for more complex motor skills since significant differences were found only for the more complex sub-parts of the SFE (neurological and midface examination) and the insertion of Bellocq’s tamponade (material preparation and catheter insertion).

Peyton’s ‘4-step Approach’ is an effective teaching method but can be time and personnel consuming since all steps have to be repeated various times by a qualified tutor. Moreover, it has been shown that trainers tend to teach practical skills with their own individual stamp [11]. Especially in a curricular setting with a high number of students and frequently changing trainers, the use of videos enables a higher standardization of the demonstration of the skills. The implementation of standardized instructional videos into Peyton’s ‘4-step Approach’ has been investigated in previous studies [10, 17, 20]. Schwerdtfeger et al. compared Peyton’s traditional ‘4-step Approach’ with a video-based ‘4-step Approach’ that replaced Steps 1 and 2 with instructional videos to teach acute clinical care of trauma patients to 313 medical students. These authors found no differences between the tested interventions [11]. Sopka et al. conducted a very similar study and compared the traditional ‘4-step Approach’ with a media-supported ‘4-step Approach’ that replaced the Steps 1 and 2 with a standardized self-produced podcast to teach external chest compressions to 220 medical students. These authors also found no significant differences between both interventions [10]. Although both of these cited studies present a high number of participants and hence, a good explanatory power, they did not compare the video-based ‘4-step Approach’ to a the often used ‘See One, Do One Approach’ and did not investigate multiple skills with different levels of difficulty. Rossettini et al. compared Peyton’s ‘4-step Approach’ to the ‘See One, Do One Approach’ in the training of manual therapy [10]. These authors found Peyton’s ‘4-step Approach’ to be more effective in the short-, medium-, and long-term. However, with 39 participants, their study lacked explanatory power and did not investigate the use of a video-based ‘4-step Approach’.

## Limitations

One shortcoming of our study is that there was no objective assessment before the intervention determining the previous experience of the individual participants. Another limitation is the performance assessment at T2 as part of a curricular and summative surgical OSCE, which might have influenced our results. A purely formative assessment would have been desirable but was not possible due to the curricular implementation of the study. The sample size (*n* = 102 students) might be a limitation to the statistical power of the sub-group analyses since it was not planned a priori.

There are also several strengths to this study. Compared to other studies, the present study was quasi-randomized controlled and blinded (at T1) carried out in a curricular “in vivo” study design with a nearly 100% participation rate and an entire cross-section of an 6th semester at an accredited medical school. Furthermore, the knowledge assessment within three points in time over a six-week span gives a comprehensive overview over the learning progress for both types of teaching interventions.

Future studies have to investigate whether the results obtained from this study can be transferred to other subjects and faculties.

## Conclusion

To our knowledge, this study is the first that compared a video-based ‘4-step Approach’ that replaced the Steps 1 to 3 with standardized instructional videos and a video-based version of the often-used ‘See One, Do One Approach’ in the mediation of multiple surgical skills with different levels of difficulty and within in a curricular setting.

We were able to show that the video-based ‘4-step Approach’ is significantly more effective than the ‘See One, Do One Approach’ in the mediation of a structured facial examination and the insertion of a Bellocq’s tamponade in the short-term, and that this holds true for the more complex parts of both skills. Furthermore, we could demonstrate that the curricular implementation of the video-based ‘4-step Approach’ was possible within a larger scale and the given time frame.

## Supplementary information

**Additional file 1: Supplement 1.** OSCE Checklist CMF – Structured Facial Examination

**Additional file 2: Supplement 2.** OSCE Checklist CMF – Placement of a Bellocq’s tamponade

## Data Availability

The datasets used and/or analyzed during the current study are available from the corresponding author on reasonable request.

## Abbreviations

OSCE: Objective Structured Clinical Examination
SFE: Structured Facial Examination
CMF: Cranio and Maxillofacial Surgery
