# Supplementary material for: Comparing video-based versions of Halsted’s ‘see one, do one’ and Peyton’s ‘4-step approach’ for teaching surgical skills: a randomized controlled trial
Source: BMC Med Educ. 2020 Jun 17;20:194. doi: 10.1186/s12909-020-02105-5 (PMC7298758; doi:10.1186/s12909-020-02105-5)
Supplement: Supplementary file 1 — Additional file 1: Supplement 1. OSCE Checklist CMF – Structured Facial Examination [file 12909_2020_2105_MOESM1_ESM.doc]

Examination

You are an intern in the emergency department.

In this cabin sits a patient who fell from the bike and fell on his head. He has no evidence of craniocerebral trauma and is vaccinated against tetanus. He also denies allergies, medication and pre-existing conditions.

1. Briefly describe pathological findings of the face on the picture presented.
2. Systematically examine Mr. Müller's head / facial area and explain what you are investigating.

You have 5 minutes time.

Student information/label

Examination

**Examiner:**  ___________________

| Examination | | Not attempted  0 | Attempted/Incomplete  1 | Correct/  Complete  2 |
| --- | --- | --- | --- | --- |
| Inspection (Describing the picture): monocle hematoma, anisocoric, nasal deviation, flattening/interruptions, swelling – if 3 and more pathologies are mentioned 2 points | |  |  |  |
| Eye | Pupils |  |  |  |
|  | Vision |  |  |  |
|  | Double vision |  |  |  |
| Nerves | Trigeminal |  |  |  |
|  | Facial |  |  |  |
| Skull | Skull cap |  |  |  |
| Midface | Orbital margin |  |  |  |
|  | Zygomatic bone |  |  |  |
|  | Le Fort I |  |  |  |
|  | Le Fort II |  |  |  |
|  | Le Fort III |  |  |  |
| Nose | Mobility |  |  |  |
|  | Inhibition of breathing |  |  |  |
|  | Septal hematoma |  |  |  |
| Lower jaw | Mandibular condyle |  |  |  |
|  | Collum/mandibular joint |  |  |  |
|  | Mandibular margin |  |  |  |
|  | Compression(chin) |  |  |  |
|  | Motility lower jaw |  |  |  |
| Intraoral | Oral inspection |  |  |  |
|  | Occlusion |  |  |  |
|  | Loose teeth |  |  |  |
|  | Opening of the mouth |  |  |  |

**Supplement 1** OSCE Checklist CMFS – Structured Facial Examination
