# Supplementary material for: Comparing video-based versions of Halsted’s ‘see one, do one’ and Peyton’s ‘4-step approach’ for teaching surgical skills: a randomized controlled trial
Source: BMC Med Educ. 2020 Jun 17;20:194. doi: 10.1186/s12909-020-02105-5 (PMC7298758; doi:10.1186/s12909-020-02105-5)
Supplement: Supplementary file 2 — Additional file 2: Supplement 2. OSCE Checklist CMF – Placement of a Bellocq’s tamponade [file 12909_2020_2105_MOESM2_ESM.doc]

Procedure

A 60-year-old patient presents to the emergency room with acute, hemodynamically relevant nosebleeds following a domestic fall.

- **Please insert a nasal (Bellocq’s) tamponade.**
- **Please explain what you are doing and what you are paying attention to.**

You have 5 minutes time.

Procedure

Student information/label

Examiner: ____________________

| Examination | Not attempted 0 | Attempted/  Incomplete  1 | Correct/  Complete  2 |
| --- | --- | --- | --- |
| Insertion of xylocaine gel (both nostrils) |  |  |  |
| Preparing a syringe with saline |  |  |  |
| Checking both catheters |  |  |  |
| Insertion of both catheters |  |  |  |
| Blocking of both catheters |  |  |  |
| Careful withdrawal both catheters |  |  |  |
| Anterior tamponade with Tampograss® under gentle pull of the catheters |  |  |  |
| Knot the ends of the tamponade |  |  |  |
| Fixation of the tamponade on the skull of the patient |  |  |  |
| On request: removal after 3 days at the latest |  |  |  |
